# Risk factors and outcomes of ventilator-associated pneumonia in COVID-19 patients: a propensity score matched analysis

**DOI:** 10.1186/s13054-021-03654-x

**Published:** 2021-07-06

**Authors:** María Martínez-Martínez, Erika P. Plata-Menchaca, Francesc X. Nuvials, Oriol Roca, Ricard Ferrer

**Affiliations:** 1 Servei de Medicina Intensiva, Hospital Universitari Vall d’Hebron, Institut de Recerca Vall d’Hebron, Passeig de la Vall d’Hebron, 119-129, 08035 Barcelona, Spain; 2 Centro de Investigación Biomédica en Red de Enfermedades Respiratorias (CIBER), Madrid, Spain

**Dear Editor**

Mechanically ventilated patients with coronavirus disease (COVID-19) may be at an increased risk of developing ventilator-associated pneumonia (VAP). Our aim was to describe the clinical characteristics, risk factors, and outcomes associated with VAP in critically ill COVID-19 patients.

In this single-center cohort study, all adult patients with laboratory-confirmed COVID-19, based on the detection of viral sequences by real-time reverse-transcription polymerase chain reaction, who needed mechanical ventilation (MV) between March 1, and April 30, 2020 were included. The local ethics committee approved this study and waived the need for informed consent due to the observational nature of the study. VAP was defined according to the current American Thoracic Society/Infectious Diseases Society of America guidelines criteria [1]. Differences between quantitative variables were assessed by Student *t*-test or Mann–Whitney *U*-test. Chi-square or Fisher´s exact tests were used to analyze categorical data. To determine which variables were independently associated with VAP, a logistic regression analysis was performed, including the variables with *p* < 0.1 in the univariate analysis. To assess the influence of VAP in outcomes and account for inter-group imbalances of baseline characteristics, we conducted a propensity score matching including those variables with *p* < 0.1 in the univariate analysis. In all cases, 1:1 matching without replacement was used with a caliper 0.2 standard deviation of logit of the propensity score and with the nearest neighbor method. In the matched cohorts, differences in the categorical variables were analyzed using McNemar’s test, and differences in continuous variables were determined using paired t-test or Wilcoxon signed-rank test, as appropriate. All statistical analyses were performed using the Stata Statistical Software 14 (StataCorp 15. College Station, TX: StataCorp LP) and R, version 3.6.3 (R project for Statistical Computing, http://www.R-project.org).

Of the 353 patients admitted to the intensive care unit (ICU) during the study period, 250 (70.8%) required invasive MV (Table 1). VAP occurred in 100 (40%) of the mechanically ventilated patients. Of these, 78 were confirmed by a positive quantitative culture and 22 were defined by clinical criteria and a purulent respiratory sample. Median time to the diagnosis of VAP was 13 (8–20) days, with only 11 developing VAP within the first 96 h of admission. Antibiotic treatment was adequate in 89% of the cases. Admission to an open ICU (contingency unit without physical barriers between patients) (odds ratio [OR], 1.85; 95% confidence interval [CI], [1.04–3.33]; *p* = 0.037), higher Sequential Organ Failure Assessment score (OR 1.55; 95% CI [1.21–2.00]; *p* = 0.001), corticosteroid treatment (OR 3.26; 95% CI [1.78–5.97]; *p* < 0.001) and treatment with tocilizumab (OR 1.85; 95% CI [1.02–3.38]; *p* = 0.044) before VAP were independently associated with VAP development. After propensity score matching, VAP was associated with a longer duration of MV and ICU stay and higher ICU mortality (Table 2).

**Table 1:** Clinical characteristics of the unmatched cohort

	All (*n* = 250)	No VAP (*n* = 150)	VAP (*n* = 100)	*p*-value
Age, median (IQR)	59 (51–67)	57 (51–67)	61.5 (52.5–67)	0.216
Gender (male), *n* (%)	173 (69.2%)	97 (64.7%)	76 (76%)	0.057
BMI > 30, median (IQR)	101 (40.4%)	63 (42%)	38 (38%)	0.528
Admission to an open ICU unit, *n* (%)	136 (54.2%)	74 (49.7%)	61 (61%)	0.078
Length of MV before onset of VAP vs. non VAP (days), median (IQR)	13 (8–20)	12 (8–19)	14 (9–21.5)	0.202
*Severity and comorbidity scores*				
APACHE II, median (IQR)	11 (8–13)	10 (7–13)	12 (9–14)	0.055
SOFA, median (IQR)	4 (3–5)	4 (3–5)	5 (3–6)	< 0.001
Charlson Comorbidity Index, median (IQR)	2 (1–3)	2 (1–3)	2 (1–3)	0.888
*COVID-19 related therapies*				
Use of prior antibiotics, *n* (%)	250 (100%)	150 (100%)	100 (100%)	1
Corticosteroids, *n* (%)*	75 (30%)	30 (20%)	45 (45%)	< 0.001
Tocilizumab, *n* (%)**	153 (60.8%)	80 (53.3%)	72 (72%)	0.003
Lopinavir–ritonavir, *n* (%)	184 (73.6%)	110 (73.3%)	74 (74%)	0.907
Remdesivir, *n* (%)	9 (3.6%)	136 (90.7%)	96 (96%)	0.110
Hydroxychloroquine, *n* (%)	232 (92.8%)	4 (2.7%)	5 (5%)	0.332
*Other ICU therapies*				
Anticoagulation, *n* (%)	86 (34.4%)	46 (30.7%)	40 (40%)	0.128
ECMO, *n* (%)	21 (8.4%)	11 (7.3%)	10 (10%)	0.456
CRRT, *n* (%)	12 (4.8%)	7 (4.7%)	5 (5.1%)	0.890
*Outcomes*				
Length of MV (days), median (IQR)	17 (10–26)	12 (8–20)	25 (20–34)	< 0.001
Length of ICU stay (days), median (IQR)	18 (12–26)	12 (8–20)	25 (20–34)	< 0.001
ICU mortality, *n* (%)	59 (23%)	24 (16%)	35 (35%)	< 0.001

APACHE II, Acute Physiologic Assessment and Chronic Health Evaluation II Score; BMI, Body Mass Index; COVID-19, coronavirus disease; CRRT*,* continuous renal replacement therapy; ECMO, Extracorporeal membrane oxygenation; ICU, Intensive Care Unit; IQR, interquartile range; MV*,* Mechanical ventilation; SOFA, Sequential Organ Failure Assessment Score; VAP, Ventilator-associated pneumonia

*Median time between corticosteroid initiation and the diagnosis of VAP was 10 days IQR (7–14)

**Tocilizumab was administered prior to ICU admission

**Table 2:** Differences in variables included in propensity score in the matched cohort

	No VAP (*n* = 78)	VAP (*n* = 78)	Difference between means (95% CI)	*p*-value
Gender (male), *n* (%)	56 (71.8)	60 (76.9)	5.1 (− 8.5 to 18.8)	0.463
Admission to an open ICU unit, *n* (%)	46 (59)	47 (60.3)	1.3 (− 14.1 to 16.7)	0.87
APACHE II, median (IQR)	10 (7–13)	11.5 (9–14)	− 0.5 (− 2 to 1)	0.758
SOFA, median (IQR)	4 (3–5)	4 (3–5)	− 0.1 (− 0.7 to 0.5)	0.759
Corticosteroids, *n* (%)	28 (35.9%)	32 (41%)	5.1 (− 10.1 to 20.4)	0.51
Tocilizumab, *n* (%)	55 (70.5%)	53 (68%)	− 2.6 (− 17 to 11.9)	0.729
Length of MV (days), median (IQR)	12 (8–20)	24 (17–33)	− 11.2 (− 17.1 to − 5.3)	< 0.001
Length of ICU stay, median (IQR)	15 (9–23)	25 (20–34)	− 11.6 (− 17.9 to − 5.3)	< 0.001
ICU Mortality, *n* (%)	11 (14.1%)	25 (32.1%)	− 17.9 (− 30.9 to − 5.0)	< 0.01

APACHE II, Acute Physiologic Assessment and Chronic Health Evaluation II Score; CI, confidence interval; ICU, intensive care unit; IQR, interquartile range; MV, Mechanical ventilation; SOFA, Sequential Organ Failure Assessment Score; VAP, Ventilator-associated pneumonia

According to the results reported, VAP is a frequent complication among mechanically ventilated COVID-19 patients [2, 3] and strongly impacts outcomes. It has been reported that COVID-19 makes patients more prone to VAP, not entirely due to the increased duration of the MV [3]. Despite the 28-day mortality benefit observed with corticosteroid use [4, 5] and controversies regarding tocilizumab use [6], both treatments were initiated prior to VAP occurrence, and often prior to ICU admission, and are independently associated with a higher risk of VAP. Limitations of the present study include its observational nature, single-center setting, and the fact that the total dose of administered steroids was not recorded. However, our findings suggest that excessive immunosuppression associated with the use of immunomodulatory drugs may facilitate VAP development in COVID-19 patients.

## Data Availability

The datasets used and analysed during the current study are available from the corresponding author on reasonable request.

## Abbreviations

COVID-19: Coronavirus disease 2019
CI: Confidence interval
ICU: Intensive care unit
MV: Mechanical ventilation
OR: Odds ratio
VAP: Ventilator-associated pneumonia
